# Complete genome sequences of *Blautia hydrogenotrophica* DSM 10507^T^ isolated from human feces and *Blautia coccoides* DSM 935^T^ isolated from mouse feces

**DOI:** 10.1128/mra.00016-24

**Published:** 2024-03-13

**Authors:** Tim Böer, Frank R. Bengelsdorf, Rolf Daniel, Anja Poehlein

**Affiliations:** 1 Genomic and Applied Microbiology and Göttingen Genomics Laboratory, Institute of Microbiology and Genetics, Georg-August University of Göttingen, Göttingen, Germany; 2 Institute of Molecular Biology and Biotechnology of Prokaryotes, University of Ulm, Ulm, Germany; Rochester Institute of Technology, Rochester, New York, USA

**Keywords:** *Blautia hydrogenotrophica*, *Blautia coccoides*, Wood-Ljungdahl pathway, acetogenic bacteria

## Abstract

We report on the closed genome sequences of the acetogen *Blautia hydrogenotrophica* S5a33^T^ (DSM 10507^T^) and of *Blautia coccoides* CLC-1^T^ (DSM 935^T^). The *B. hydrogenotrophica* S5a33^T^ genome harbors a chromosome (3,590,609 bp) and a plasmid (7,176 bp). The *B. coccoides* CLC-1^T^ genome consists of a single chromosome (6,097,890 bp).

## ANNOUNCEMENT

The strains *Blautia hydrogenotrophica* S5a33^T^ (DSM 10507^T^), *Blautia schinkii* B^T^ (DSM 10518^T^), *Blautia producta* U-1 (DSM 3507), and *Blautia coccoides* GA-1 were described to grow autotrophically with H_2_ + CO_2_ using the Wood-Ljungdahl pathway (1–4). However, with the exception of *B. producta,* high-quality type strain genomes were not available for acetogenic bacteria of the genus *Blautia*. Here, we report complete genome sequences of the type strains *B. hydrogenotrophica* S5a33^T^ (DSM 10507^T^, formerly *Ruminococcus hydrogenotrophicus*) isolated from human feces and *B. coccoides* CLC-1^T^ (DSM 935^T^, formerly *Clostridium coccoides*) isolated from mouse feces (1, 5). Both strains were cultivated in 10 mL of DSM 311c medium as listed by the German Collection of Microorganisms and Cell Cultures (DSMZ, Braunschweig, Germany) under anaerobic conditions. Cells were inoculated from lyophilized stock cultures from the DSMZ in an anaerobic chamber and incubated in Hungate tubes at 35°C for 12 h without shaking. Cells were harvested by centrifugation at 18,000 × *g* for 5 min. Illumina and Nanopore sequencing were performed with separately grown cell cultures. DNA was isolated by using the Master-Pure Complete DNA and RNA purification kit (Epicentre, Madison, WI, USA) following the instructions for cell samples. Illumina sequencing was performed by preparation of Illumina sequencing libraries using the Nextera XT DNA sample preparation kit and a MiSeq system with v3 chemistry (2 × 300  bp, 600 cycles) following the instructions of the manufacturer (Illumina, San Diego, CA, USA). Long-read sequencing was performed by preparation of Nanopore sequencing libraries with 1.5 µg high-molecular-weight DNA using the ligation sequencing kit 1D (SQK-LSK109) and the native barcode expansion kit (EXP-NBD114) as recommended by the manufacturer (Oxford Nanopore Technologies, Oxford, UK). Nanopore sequencing was conducted for 72 h with the MinION device Mk1B, a SpotON flow cell R9.4.1, and the MinKNOW software (v21.06.13) following the instructions of the manufacturer (Oxford Nanopore Technologies). Default parameters were used for all software unless otherwise specified. Demultiplexing and base calling of Nanopore sequencing data were performed with the Guppy software in HAC mode (v.5.0.16). Long-read *de novo* genome assemblies were obtained by following the described Trycycler workflow and parameters for bacterial genome assemblies by Wick et al. (6). The following programs were used for genome assemblies: fastp (v0.23.2), trimmomatic (v0.39; LEADING: 3, TRAILING: 3, SLIDINGWINDOW:4:15, MINLEN:50), Porechop (v0.2.4), Filtlong (v0.2.1), Trycycler (v0.5.3), Flye (v2.9.1), Canu (v2.2), and Raven (v1.8.1) (7–12). Flye, Canu, and Raven assemblies were combined to a single consensus sequence with Trycycler, and the consensus sequence was polished with long-read data using Medaka (v1.5.0) and finally polished with Illumina sequences using Polypolish (v0.5.0) (13), resulting in a circular chromosome in both cases. Genome annotations were performed with Prokka (v1.14.6) and quality assessment of the final genome assemblies was conducted with CheckM2 (v1.0.2) (14, 15). Details of sequencing and genome statistics of *B. hydrogenotrophica* and *B. coccoides* obtained in both cases are summarized in Table 1.

**TABLE 1:** Sequencing statistics and genome features of *B. hydrogenotrophica* DSM 10507^T^ and B. coccoides DSM 935^T^

Feature	*B. hydrogenotrophica* DSM 10,507^T^	*B. coccoides* DSM 935^T^
Chromosome size (bp)	3,590,609	6,097,890
Number of Illumina reads (250 bp)	804,958	646,392
Number of Nanopore reads/mean length (bp)	80,177/4,543	55,589/7,066
Chromosome mean coverage (Illumina/Nanopore)	103/96	56/63
Plasmid size (bp)	7,176	–
Plasmid mean coverage (Illumina/Nanopore)	2,894/1,418	–
GC content (%)	45	46
Genes	3,465	5,466
CDS	3,387	5,391
Functional proteins	1,869	2,803
Hypothetical proteins	1,518	2,588
rRNAs (5S, 16S, 23S)	8 (4, 4, 4)	10 (5, 5, 5)
tRNAs	69	64
tmRNAs	1	1
CheckM2:		
Completeness score (%)	98.25	100
Contamination score (%)	0.47	3.88

## Data Availability

Genome sequences were deposited under the GenBank accession numbers CP136423 and CP136424 (*B. hydrogenotrophica*) and CP136422 (*B. coccoides*). Raw read data were deposited in the NCBI Sequence Read Archive (SRA) under the accession numbers SRR27175288 (Illumina) and SRR27175321 (Nanopore) for *B. coccoides* and SRR27175281 (Illumina) and SRR27183504 (Nanopore) for *B. hydrogenotrophica*.
